# Facile Functionalization of Electrospun Poly(ethylene-*co*-vinyl alcohol) Nanofibers via the Benzoxaborole-Diol Interaction

**DOI:** 10.3390/polym8020041

**Published:** 2016-02-05

**Authors:** Yohei Kotsuchibashi, Mitsuhiro Ebara

**Affiliations:** 1 International Center for Young Scientists (ICYS) and International Center for Materials Nanoarchitectonics (WPI-MANA), National Institute for Materials Science (NIMS), 1-1 Namiki, Tsukuba, Ibaraki 305-0044, Japan; 2 Biomaterials Unit, WPI-MANA, NIMS, 1-1 Namiki, Tsukuba, Ibaraki 305-0044, Japan; EBARA.Mitsuhiro@nims.go.jp; 3 Graduate School of Industrial Science and Technology, Tokyo University of Science, 6-3-1 Niijuku, Katsushika, Tokyo 125-8585, Japan

**Keywords:** benzoxaborole, boroxole, poly(ethylene-*co*-vinyl alcohol), nanofiber, electrospinning, gel, smart polymer, stimuli response, pH response

## Abstract

A facile functionalization method of poly(ethylene-*co*-vinyl alcohol) (EVOH) nanofiber meshes was demonstrated by utilizing the benzoxaborole-diol interaction between EVOH and benzoxaborole-based copolymers (BOP). EVOH and BOP were firstly mixed to prepare the quasi-gel-state solution with enough viscosity for electro-spinning. The fiber morphology was controlled via changing the mixing ratio of EVOH and BOP. The prepared EVOH/BOP nanofiber mesh showed good stability in aqueous solution. Over 97% of the nanofibers remained after the immersion test for 24 h in acid or alkali aqueous solutions without changing their morphology. Temperature and pH-responsive moieties were copolymerized with BOP, and cationic dye was easily immobilized into the nanofiber mesh via an electrostatic interaction. Therefore, the proposed functionalization technique is possible to perform on multi-functionalized molecule-incorporated nanofibers that enable the fibers to show the environmental stimuli-responsive property for the further applications of the EVOH materials.

## 1. Introduction

Poly(ethylene-*co*-vinyl alcohol) (EVOH) has been one of the best known flexible thermoplastic materials and used in a wide range of fields, such as food packaging, funnel tanks, and medical applications for the high gas-barrier and biocompatibility [1,2,3]. The fabrication of EVOH materials has been customized dependent on the choice of applications, such as films [4], (nano)particles [5], porous materials [6] and (nano)fibers [7,8].

Moreover, these EVOH materials have been modified with molecules/polymers to enhance their functionalities. The modification, however, is limited due to the multi-steps of the reaction processes. Therefore, a simple modification system for EVOH materials has been focused on recently. Zhou *et al.* prepared a phosphorylcholine-modified EVOH in two reaction steps to prevent nonspecific protein adsorption [9]. Zhu *et al.* modified chelating groups on EVOH nanofibers via a three-step treatment, the modified nanofibers were used for a selective protein separation system [10]. Plasma treatment achieves a one-step modification for EVOH materials. By the treatment, however, the EVOH composition was changed via the newly-generated groups, such as carboxylic acid. The conversion also leads to a change of the physicochemical properties of the original EVOH [11]. As other one-step modifications for EVOH materials, there is a blending method with hydrophobic polymers via the hydrophobic-hydrophobic interaction [12]. The mechanical properties of EVOH nanofibers were strengthened by mixing with polymers, such as polypropylene (PP) [13], poly(lactic acid) (PLA) [14], cellulose [15] and Nylon 6/12 [16]. However, it is difficult to obtain stable EVOH blend materials with a hydrophilic polymer in aqueous solution because of their weak interactions. Stable EVOH and hydrophilic polymer blends in aqueous solution were reported using multi-step treatments, such as a polymerization of hydrophilic polymers from the EVOH surface [17,18] and a cross-linking method after the blend process [19]. Moreover, a copolymerization of ethylene, vinyl acetate (precursor of vinyl alcohol) and hydrophilic monomers is expected to obtain a modified EVOH with hydrophilic units. However, typical (meth)acrylate and (meth)acrylamide monomers have different reaction ratios with ethylene and vinyl acetate due to their structure, which leads to low yields and a gradient composition [20]. Therefore, it remains a challenge to propose a simple EVOH function system. To achieve this system, we focused on a dynamic covalent chemistry [21,22].

Recently, we have reported a hydrogel gel system using reversible covalent bonding between benzoxaborole-based temperature-responsive copolymers and glyco-based copolymers [23,24]. The benzoxaborole unit can reversibly combine with the *cis*-diol in the glyco-based copolymers. The mixed hydrogels displayed temperature-responsive, pH-responsive and glucose-responsive properties. Moreover, a photo-acid generator of 2-nitrobenzaldehyde (2-NBA) that possesses a proton-release by UV irradiation [25] was encapsulated into the hydrogel. The UV irradiation caused the disintegration of the hydrogel structure in the exposed region, resulting in the local pH decrease. The benzoxaborole units have also been applied in biomedical fields, such as the selective binding with the Thomsen–Friedenreich (TF)-antigen disaccharide, delivery of a protein toxin in the cytosol, neutralization of human immunodeficiency virus (HIV) and antitrypanosomal agent [26,27,28,29,30].

**Figure 1:**
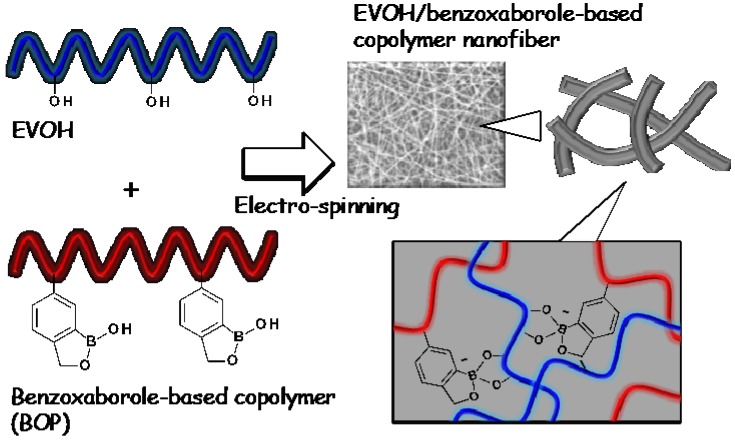
Schematic representation of modified EVOH nanofiber with benzoxaborole-based copolymers (BOPs).

In this study, EVOH was mixed with the water-soluble benzoxaborole-based copolymers (BOPs) in organic solvent and was found to show a gelation via a benzoxaborole-diol interaction. The functionalized EVOH nanofiber meshes with BOP were prepared by electro-spinning using the mixed viscous solution (Figure 1). The nanofiber meshes had a stable cross-linked structure in aqueous solution due to the reversible covalent bonding. Using this simple method, the EVOH nanofibers were successfully functionalized with stimuli-responsive copolymers. To the best of our knowledge, this is the first report on functionalized EVOH materials with BOPs. The stability and functionality of the EVOH/BOP nanofiber meshes were investigated in various solution conditions.

## 2. Experimental Section

### 2.1. Material

Five-methacrylamido-1,2-benzoxaborole (MAAmBO) was synthesized and purified according to the protocol given [23,24]. One-pyrenemethyl methacrylate (PyMA) was purified by recrystallization from ethanol. Two-(2-methoxyethoxy)ethyl methacrylate (MEO_2_MA), oligo(ethylene glycol) methacrylate (OEGMA, *M*_n_ = 475 g/mol) and acrylic acid (Ac) were purchased from Sigma–Aldrich (St. Louis, MO, USA) and purified by passing through a basic alumina column. EVOH (EVAL E105A, 44 mol % ethylene) was kindly supplied from KURARAY (Okayama, Japan). All other chemicals and solvents were used as received. Distilled water used in this study was purified with a Millipore Milli-Q system.

### 2.2. Preparation of Benzoxaborole-Based Copolymers

Reversible addition-fragmentation chain transfer (RAFT) polymerization was employed to synthesize copolymers with a narrow molecular weight distribution. The polymerization methods of BOPs are described in the Supplementary Materials. Briefly, MEO_2_MA (540 mg, 2.87 mmol), OEGMA (908 mg, 1.91 mmol), MAAmBO (116 mg, 0.53 mmol), 4-cyanopentanoic acid dithiobenzoate (CTP) (7.42 mg, 2.66 × 10**^−^**^2^ mmol) and 4,4’-azobis-4-cyanovaleric acid (ACVA) (2.98 mg, 1.06 × 10**^−^**^2^ mmol) ([MEO_2_MA]_0_/[OEGMA]_0_/[MAAmBO]_0_/[CTP]_0_/[ACVA]_0_ = 108/72/20/1/0.4) were dissolved in 4 mL of methanol. After degassing with nitrogen gas for 30 min, the mixture was allowed to polymerize for 24 h at 60 °C. The resulting P(MEO_2_MA-*co*-OEGMA-*co*-MAAmBO) was purified by dialysis against ethanol and acetone and was dried under reduced pressure. The preparation conditions of other BOPs are shown in the Supplementary Materials.

### 2.3. Preparation of EVOH/BOP Nanofiber Meshes

BOPs were dissolved in 1,1,1,3,3,3-hexafluoroisopropanol (HFIP) at room temperature. Electro-spinning was performed using a Nanon-01A (MECC, Fukuoka, Japan). Spinning parameters were kept constant at a 25-kV applied voltage, a 1.0-mL/h solution flow rate, a 15-cm working distance and a 25-gauge pointed needle [8]. The fibers were electrospun onto a sheet of aluminum foil on a stationary plate collector. The fibers were directly extracted from the foil.

### 2.4. Stability Tests

Nanofiber meshes of EVOH and BOPs (P(MEO_2_MA-*co*-MAAmBO-*co*-PyMA) or P(MEO_2_MA-*co*-PyMA)) were prepared for stability tests in various solution conditions (pH 2/37 °C, pH 2/4 °C, pH 12/37 °C and pH 12/4 °C). The PyMA units were used to detect the amount of released BOPs from the nanofibers. The BOP and EVOH were dissolved in HFIP at 2 and 7 wt % concentrations, respectively. Each 2.5-mL polymer solution was mixed and was kept at least 1 h to reach the equilibrium before electro-spinning. Nanofiber meshes were cut (2.70–3.79 mg) as a square sample and were added to pH 2 (0.01 N HCl) or pH 12 (0.01 N NaCl) solution to be the same concentration (0.3 wt %, e.g., 3 mg of fiber sample was added to 1 mL solution). After 24 h, all nanofiber samples were washed with Milli-Q and were dried under atmospheric pressure. The morphology and weight of the nanofiber meshes were measured. Each nanofiber sample was measured at 3 different places (*N* = 3).

### 2.5. Characterizations

^1^H NMR spectra of copolymers were taken with a JNM-GSX300 spectrometer operating at 300 MHz (JEOL, Tokyo, Japan) to confirm successful synthesis and to determine the chemical composition of the synthesized copolymers. Molecular weight and polydispersity of the synthesized copolymers were determined by gel permeation chromatography (GPC) at 40 °C (DMF, including 10 mM LiBr, 1 mL/min) with a TOSOH TSK-GEL a-2500 and a-4000 and (Tosoh, Tokyo, Japan) connected to an RI-2031 refractive index detector (JASCO International Co. Ltd., Tokyo, Japan). Transmittance of a copolymer solution at 500 nm was continuously recorded at a heating rate of 1.0 °C/min by a UV–Vis spectrometer V-550 (JASCO International Co. Ltd., Tokyo, Japan) to measure the lower critical solution temperature (LCST). Synthesized copolymers were dissolved in aqueous solution at the given concentration. LCSTs of copolymers were determined at 50% transmittance. Fluorescence spectra were recorded using a fluorescence spectrometer F-2500 (Hitachi High-Technologies Corporation, Tokyo, Japan). The morphologies of nanofiber meshes were observed using a NEO-Scope JCM-5000 SEM (JEOL, Tokyo, Japan).

## 3. Results and Discussion

### 3.1. Preparation of Benzoxaborole-Based Copolymers

**Figure 2:**
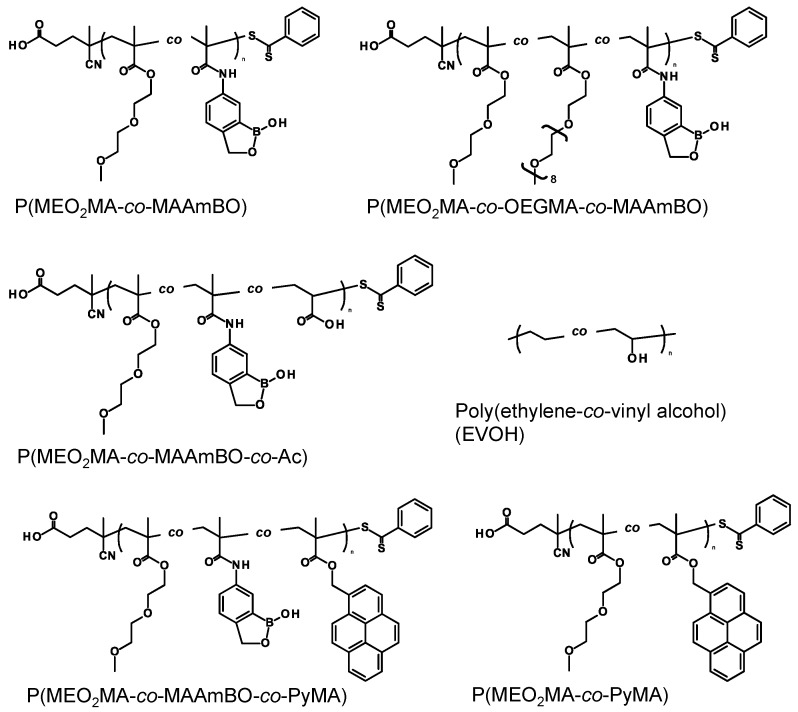
Chemical structure of BOPs and EVOH. MEO_2_MA, two-(2-methoxyethoxy)ethyl methacrylate; MAAmBO, five-methacrylamido-1,2-benzoxaborole; OEGMA, oligo(ethylene glycol) methacrylate; Ac, acrylic acid; PyMA, one-pyrenemethyl methacrylate.

The BOPs were polymerized by the reversible addition-fragmentation chain transfer (RAFT) polymerization. Boronic acids and their esters can reversibility interact with the *cis*-diol group. The interaction between phenylboronic acid (PBA) and glucose has been utilized as a trigger for an insulin release system that was incorporated into newly-designed polymeric materials [31,32,33]. Recently, Hall and co-workers reported that the benzoxaborole molecule showed a higher affinity than boronic acid toward saccharides in phosphate-buffered saline (PBS) [34]. The high affinity comes from the relatively low pKa of the benzoxaborole (pK_a_ 7–8) as compared to that of boronic acid (pK_a_ 8–9) [35,36]. In this study, benzoxaborole-based monomers were copolymerized with functional monomers for the functionalization of EVOH nanofiber meshes (Figure 2). The compositions of the BOPs are summarized in Table 1. The copolymers, P(MEO_2_MA-*co*-MAAmBO), P(MEO_2_MA-*co*-OEGMA-*co*-MAAmBO) and P(MEO_2_MA-*co*-MAAmBO-*co*-Ac) were prepared. The monomers of MEO_2_MA and OEGMA were selected in order to control the temperature-responsive properties, *i.e.*, lower critical solution temperature (LCST). The LCSTs of P(MEO_2_MA) and P(OEGMA) homopolymers were 28 and 90 °C, respectively [37]. The Ac monomer with carboxylic acid functionality was used to donate the pH-responsive property to the BOPs. Moreover, the anionic charge can interact with cationic molecules via electrostatic interactions. The compositions (mol %) of the BOPs were measured using ^1^H NMR and are shown at the right side of monomer unit (P(MEO_2_MA_91.0_-*co*-MAAmBO_9.0_), P(MEO_2_MA_56.5_-*co*-OEGMA_38.2_-*co*-MAAmBO_5.3_) and P(MEO_2_MA_86.7_-*co*-MAAmBO_4.8_-*co*-Ac_8.5_). Molecular weight (*M*_n_) and *M*_w_/*M*_n_ of the BOPs were calculated from GPC measurement. However, broad GPC peaks were observed in all BOPs (40 °C, DMF including 10 mM LiBr, 1 mL/min). This might be an adsorption between MAAmBO units and the filler of the GPC column. Therefore, the benzoxaborole units were protected with 1,4-butanediol to prevent the adsorption [38], and the molecular weights were measured using the same GPC conditions. The molecular weights were smaller than those of their targeted monomer units (200 units) (P(MEO_2_MA_91.0_-*co*-MAAmBO_9.0_): *M*_n_ = 3000 g/mol, *M*_w_/*M*_n_ = 1.26; P(MEO_2_MA_56.5_-*co*-OEGMA_38.2_-*co*-MAAmBO_5.3_): *M*_n_ = 6000 g/mol, *M*_w_/*M*_n_ = 1.80; and P(MEO_2_MA_86.7_-*co*-MAAmBO_4.8_-*co*-Ac_8.5_): *M*_n_ = 2300 g/mol, *M*_w_/*M*_n_= 1.32). The estimated low molecular weights might come from the unprotected benzoxaborole units and the solubility change that occurred due to the protecting groups. The stimuli-responsive properties of BOPs were measured using the transmittance change as a function of temperature (Figures S1 and S2). The LCST of the P(MEO_2_MA_91.0_-*co*-MAAmBO_9.0_) was around 18.3 °C at pH 2 (0.01N HCl_aq._) (Figure S1A). The hydrophobic MAAmBO units led to a decrease in the LCST. On the other hand, at pH 12 (0.01 N NaOH_aq._), the transmittance change of P(MEO_2_MA_91.0_-*co*-MAAmBO_9.0_) disappeared due to the anionic charged MAAmBO units, resulting in the electrostatic repulsion. The LCST of P(MEO_2_MA_56.5_-*co*-OEGMA_38.2_-*co*-MAAmBO_5.3_) was observed at 63.5 °C at pH 2 due to the hydrophilic OEGMA units, and no transmittance change was observed at pH 12 (Figure S1B). These results suggested that the LCST of the BOPs could be easily controlled by the copolymerized monomer compositions. P(MEO_2_MA_86.7_-*co*-MAAmBO_4.8_-*co*-Ac_8.5_) had the carboxylic acid groups as the pH-responsive units. At pH 2, the LCST was 17.6 °C. On the other hand, at pH 12, no transmittance change was observed due to the electrostatic repulsion of both MAAmBO and Ac units (Figure S1C).

**Table 1:** Characterization of BOPs. LCST, lower critical solution temperature.

Copolymer	In Copolymer (mol%) ^a^					*M*_n_^b^ (g/mol)	*M*_w_/*M*_n_^b^ (–)	LCST (°C) ^c^	
	MEO_2_MA	MAAmBO	OEGMA	Ac	PyMA			pH 2 ^d^	pH 12 ^e^
P(MEO_2_MA_91.0_-*co*-MAAmBO_9.0_)	91.0	9.0	–	–	–	3000	1.26	18.3	no LCST ^f^
P(MEO_2_MA_56.5_-*co*-OEGMA_38.2_-*co*-MAAmBO_5.3_)	56.5	5.3	38.2	–	–	6000	1.80	63.5	no LCST ^f^
P(MEO_2_MA_86.7_-*co*-MAAmBO_4.8_-*co*-Ac_8.5_)	86.7	4.8	–	8.5	–	2300	1.32	17.6	no LCST ^g^
P(MEO_2_MA_93.9_-*co*-MAAmBO_5.2_-*co*-PyMA_0.9_)	93.9	5.3	–	–	0.9	2600	1.59	15.2	no LCST ^f^
P(MEO_2_MA_99.0_-*co*-PyMA_1.0_)	99.0	–	–	–	1.0	12,500	1.31	21.3 (pH 7.4 PBS)	

^a^ The copolymer compositions were calculated by ^1^H NMR; ^b^ determined by gel permeation chromatography (GPC) using 10 mM LiBr DMF; ^c^ LCSTs were determined at the temperature with 50% of transmittance; ^d^ 0.01 N-HCl_aq._ was used; ^e^ 0.01 N-NaOH_aq._ was used. No transmittance change was observed by ^f^ 90 and ^g^ 70 °C.

### 3.2. Preparation of EVOH/BOP Nanofiber Meshes by Electro-Spinning

**Figure 3:**
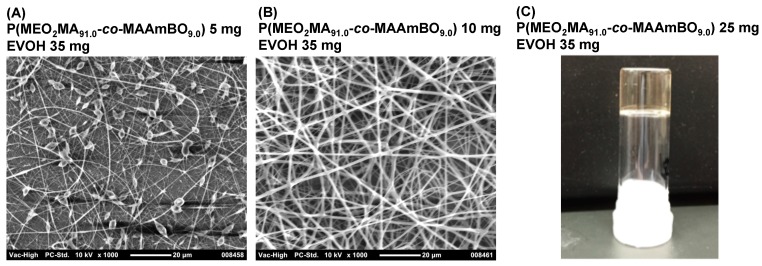
SEM image of electro-spinning samples of EVOH (35 mg) and (**A**) P(MEO_2_MA_91.0_-*co*-MAAmBO_9.0_) (5 mg) and (**B**) P(MEO_2_MA_91.0_-*co*-MAAmBO_9.0_) (10 mg) in 1 mL 1,1,1,3,3,3-hexafluoroisopropanol (HFIP). (**C**) Gel formation of EVOH (35 mg) and P(MEO_2_MA_91.0_-*co*-MAAmBO_9.0_) (25 mg) in 1 mL HFIP.

**Figure 4:**
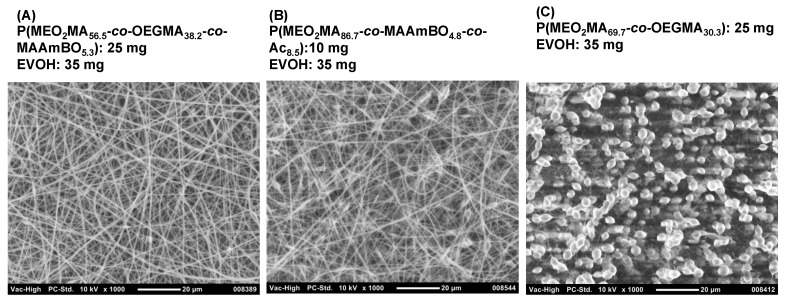
SEM images of electro-spinning samples: (**A**) P(MEO_2_MA_56.5_-*co*-OEGMA_38.2_-*co*-MAAmBO_5.3_) (25 mg) and EVOH (35 mg), (**B**) P(MEO_2_MA_86.7_-*co*-MAAmBO_4.8_-*co*-Ac_8.5_) (10 mg) and EVOH (35 mg) and (**C**) P(MEO_2_MA_69.7_-*co*-OEGMA_30.3_) (25 mg) and EVOH (35 mg) in 1 mL HFIP.

BOPs can interact with OH groups in the EVOH. Kikuchi *et al.* reported a hydrogel that was composed of a boronic acid-based copolymer and a water-soluble poly(vinyl alcohol) (PVA) [39]. We have prepared hydro(nano)gels consisting of glyco-based copolymers and temperature-responsive P(*N*-isopropylacrylamide (NIPAAm)-*co*-MAAmBO)s [23,24]. The water-soluble glyco-based copolymers were mixed with P(NIPAAm-*co*-MAAmBO) in aqueous solution. However, EVOH is a water-insoluble copolymer, which means a good solvent for both copolymers has to be selected for the electro-spinning method. HFIP was selected as a good solvent. The EVOH solution (35 mg, 500 μL HFIP) was mixed with P(MEO_2_MA_91.0_-*co*-MAAmBO_9.0_) at different concentrations, and the mixed solutions were used in electro-spinning (Figure 3). At a low concentration of P(MEO_2_MA_91.0_-*co*-MAAmBO_9.0_) (5 mg, 500 μL HFIP), nanofibers and aggregated particles were observed (Figure 3A). It has been observed that electro-spinning with a low viscous solution can result in aggregated particles [8]. When the concentration was increased using P(MEO_2_MA_91.0_-*co*-MAAmBO_9.0_) (10 mg, 500 μL HFIP), fine nanofiber formation was observed (Figure 3B). The interaction between MAAmBO and OH units led to the increase of the viscosity, resulting in the increasing superficial molecular weight. At high concentrations of P(MEO_2_MA_91.0_-*co*-MAAmBO_9.0_) (25 mg, 500 μL HFIP), gel formation was observed, which could not be from electro-spinning (Figure 3C). Fine nanofibers were also obtained from other BOPs. Figure 4A,B shows the nanofiber structures of EVOH/P(MEO_2_MA_56.5_-*co*-OEGMA_38.2_-*co*-MAAmBO_5.3_) (25 mg, 500 μL HFIP) and EVOH/P(MEO_2_MA_86.7_-*co*-MAAmBO_4.8_-*co*-Ac_8.5_) (10 mg, 500 μL HFIP), respectively. High MAAmBO content led to the need for a small amount of copolymers for the nanofiber structure. These results suggested that the nanofiber structures were affected by the MAAmBO contents, polymer concentrations and their mixture ratios. For example, imperfect nanofiber formation (with particle aggregation) was observed in the mixture of P(MEO_2_MA_69.7_-*co*-OEGMA_30.3_) (25 mg) (*M*_n_ = 25,400 g/mol, *M*_w_/*M*_n_ = 1.28 [39]) (Figure 4C). The P(MEO_2_MA_69.7_-*co*-OEGMA_30.3_) has no MAAmBO unit and cannot interact with EVOH. The lack of increase in viscosity led the imperfect nanofiber formation.

### 3.3. Stability Test of EVOH/BOP Nanofiber Meshes

**Figure 5:**
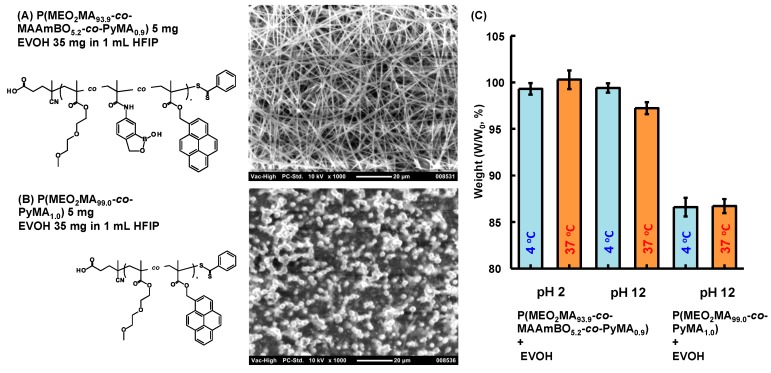
SEM images of electro-spinning samples: (**A**) P(MEO_2_MA_93.9_-*co*-MAAmBO_5.2_-*co*-PyMA_0.9_) (5 mg) and EVOH (35 mg); and (**B**) P(MEO_2_MA_99.0_-*co*-PyMA_1.0_) and EVOH (35 mg) in 1 mL HFIP. (**C**) Weight loss ((weight after immersion test, *W*)/(weight before immersion test, *W*_0_) × 100 %) at various immersion conditions for 24 h.

Multi-step organic reactions are needed to modify molecules in EVOH materials. By simple mixing, the EVOH nanofiber mesh was easily modified with the BOPs. As the copolymers, P(MEO_2_MA_93.9_-*co*-MAAmBO_5.2_-*co*-PyMA_0.9_) and P(MEO_2_MA_99.0_-*co*-PyMA_1.0_) were selected (Table 1 and Figure 2). The fluorescent molecule of PyMA can trace the morphology change of a polymer, chain and the fluorescent intensity can also be an indicator for cell biology [40]. To prevent the LCST change via the hydrophobic PyMA units, the PyMA contents in the copolymers were adjusted less than 1 mol %. In fact, the LCST of P(MEO_2_MA_99.0_-*co*-PyMA_1.0_) was 21.3 °C (Table 1 and Figure S2) in pH 7.4 PBS, which was similar to that of the P(MEO_2_MA) homopolymer (LCST: 23 °C in PBS [40]). The stability of the nanofiber meshes of EVOH/BOP was measured at various solution conditions. EVOH/P(MEO_2_MA_93.9_-*co*-MAAmBO_5.2_-*co*-PyMA_0.9_) nanofiber meshes were immersed for 24 h in pH 2 (HCl_aq._) and 12 (NaOH_aq._) at different solution temperatures (4 and 37 °C). Figure 5A shows the SEM images of EVOH/P(MEO_2_MA_93.9_-*co*-MAAmBO_5.2_-*co*-PyMA_0.9_) nanofiber meshes. After immersion treatment, there was no outward morphology change on the nanofiber structure (Figure S3A,B). At the same immersion conditions, particle aggregation was observed in the modified EVOH nanofiber with P(MEO_2_MA_99.0_-*co*-PyMA_1.0_) (Figure 5B and Figure S3C–F). The weight loss of the nanofiber meshes after immersion is shown in Figure 5C. In the P(MEO_2_MA_93.9_-*co*-MAAmBO_5.2_-*co*-PyMA_0.9_) at pH 12, the weight losses were small at both 4 and 37 °C; over 97% of copolymers remained in the nanofiber structure. The high stability is a result of the interaction between EVOH and MAAmBO units. Interestingly, at pH 2, the weight losses were also small at both 4 and 37 °C (over 98% of copolymer remained in the nanofiber). The small weight loss might be due to three reasons: (1) polymeric entanglement; (2) hydrophobic-hydrophobic interaction; and (3) boroxole and *cis*-diol interaction at acidic condition [35,36]. On the other hand, the weight losses of P(MEO_2_MA_99.0_-*co*-PyMA_1.0_) that cannot interact with EVOH via the covalent bonding were 86.6 and 86.7% at 4 and 37 °C, respectively. Moreover, after the immersion test, the supernatant liquid was also measured using a fluorescent detector to estimate the released amount of the P(MEO_2_MA_99.0_-*co*-PyMA_1.0_) from the nanofiber meshes. The standard straight line (fluorescent intensity *vs*. concentration) was made from P(MEO_2_MA_99.0_-*co*-PyMA_1.0_) (*R*^2^ = 0.991). The calculated release of P(MEO_2_MA_99.0_-*co*-PyMA_1.0_) was 5% at 4 °C. There was a difference in the calculated release between the weight loss measurements and the concentration determined by fluorescent intensity. The supernatant liquid might have a very small amount of EVOH, which led to a different external environment as compared to that of free P(MEO_2_MA_99.0_-*co*-PyMA_1.0_), resulting in the discrepancy with the standard straight line.

### 3.4. Controlled Adsorption and Desorption of Cationic Dye on Anionic EVOH/Benzoxaborole Nanofiber Meshes

**Figure 6:**
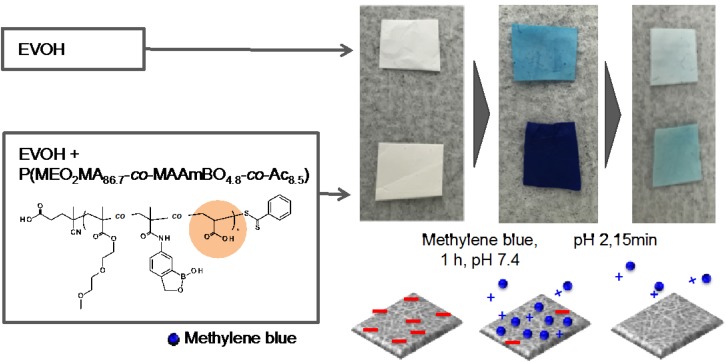
pH-responsive dye tests of the EVOH nanofiber and EVOH/P(MEO_2_MA_86.7_-*co*-MAAmBO_4.8_-*co*-Ac_8.5_) nanofiber using methylene blue solution. Blue and red colors in the illustration are cationic methylene blue and anionic Ac units, respectively.

The nanofiber of EVOH/P(MEO_2_MA_86.7_-*co*-MAAmBO_4.8_-*co*-Ac_8.5_) was utilized for its pH-responsive property in a molecule catch and release system. The pH-responsive units of the P(MEO_2_MA_86.7_-*co*-MAAmBO_4.8_-*co*-Ac_8.5_) occur due to the carboxylic acid located in the Ac unit. At pH 2, the LCST was 17.6 °C. On the other hand, at pH 12, no transmittance change was observed due to the electrostatic repulsion of both MAAmBO and Ac units (Table 1). Therefore, the surface properties of functionalized EVOH nanofiber mesh will be altered at different pH via the mixed P(MEO_2_MA_86.7_-*co*-MAAmBO_4.8_-*co*-Ac_8.5_). The anionic EVOH/P(MEO_2_MA_86.7_-*co*-MAAmBO_4.8_-*co*-Ac_8.5_) nanofiber mesh was immersed in cationic methylene blue solution at pH 7.4 PBS (Figure 6). The anionic charged nanofiber mesh was expected to adsorb the methylene blue strongly via an electrostatic interaction as compared to that of only EVOH nanofiber mesh. In fact, the EVOH/P(MEO_2_MA_86.7_-*co*-MAAmBO_4.8_-*co*-Ac_8.5_) nanofiber mesh strongly adsorbed the blue dye more than that of the EVOH nanofiber mesh. The dyed nanofiber meshes were immersed in pH 2 solution to release the methylene blue via the pH-responsive property of the Ac units in the P(MEO_2_MA_86.7_-*co*-MAAmBO_4.8_-*co*-Ac_8.5_). After 15 min, the blue color clearly turned light. In other words, the functionalized EVOH nanofiber mesh achieved a controlled molecule adsorption and desorption depending on the solution pH. The nanofibers were reusable for the controlled adsorption/desorption of methylene blue (Figure S4). These results suggested that the pH-responsive properties of BOPs were imparted into the electro-spun EVOH nanofiber meshes. This simple functionalization system will be a useful tool in expanding the application field of EVOH (nano)materials.

## 4. Conclusions

In conclusion, we proposed a facile functionalization method of EVOH nanofiber meshes using the BOPs via their reversible benzoxaborole-diol interaction. The mixture solution of EVOH and BOPs was prepared in HFIP for electro-spinning; it was found that the benzoxaborole-diol interaction occurred in the organic solvent. The interaction resulted in increased viscosity, which supported the fabrication of a fine nanofiber structure. The structure of the nanofiber mesh was strongly affected by the benzoxaborole content, polymer concentration and the mixture ratios. Particles and bulk gels were also formed depending on the preparation conditions. The modified EVOH nanofiber meshes showed a high stability in acid or alkali aqueous solutions. After a 24-h immersion test, over 97% of EVOH and BOP remained within the nanofiber mesh. According to the SEM images, there was no morphology change in the nanofiber structure after the immersion test. Moreover, the EVOH nanofiber mesh was also functionalized with BOP having pH-responsive COOH groups. The anionic charged nanofiber mesh was strongly dyed by methylene blue due to the electrostatic interaction as compared to that of the EVOH nanofiber mesh in pH 7.4. The dye was rapidly released from the fiber mesh in acidic condition. This simple and effective functionalization system for EVOH (nano)materials could lead to an expansion of their applications into new fields.

## Supplementary Materials

Supplementary materials can be found at www.mdpi.com/2073-4360/8/2/41/s1.
