# Supplementary material for: Facile Functionalization of Electrospun Poly(ethylene-co-vinyl alcohol) Nanofibers via the Benzoxaborole-Diol Interaction
Source: Polymers (Basel). 2016 Feb 5;8(2):41. doi: 10.3390/polym8020041 (PMC6432579; doi:10.3390/polym8020041)
Supplement: Supplementary file 1 [file polymers-08-00041-s001.pdf]

Supplementary Materials: Facile Functionalization of Electrospun Poly(ethylene-*co*-vinyl alcohol) Nanofibers via the Benzoxaborole-Diol Interaction

Yohei Kotsuchibashi and Mitsuhiro Ebara

1. Preparation of P($MEO_2MA_{91.0}$-*co*-$MAAmBO_{9.0}$)

MEO_2MA (900 mg, 4.78 mmol), MAAmBO (116 mg, 0.53 mmol), 4-cyanopentanoic acid dithiobenzoate (CTP) (7.42 mg, 2.66 × 10^{-2} mmol) and 4,4′-azobis-4-cyanovaleric acid (ACVA) (2.98 mg, 1.06 × 10^{-2} mmol) ($[MEO_2MA]_0/[MAAmBO]_0/[CTP]_0/[ACVA]_0$ = 180/20/1/0.4) were dissolved in 4 mL of methanol. After degassing with nitrogen gas for 30 min, the mixture was allowed to polymerize for 24 h at 60 °C. The resulting P(MEO_2MA-*co*-MAAmBO) was purified by dialysis against ethanol and acetone and was dried under reduced pressure.

2. Preparation of P(MEO2MA86.7-*co*-MAAmBO4.8-*co*-Ac8.5)

MEO2MA (850 mg, 4.52 mmol), MAAmBO (116 mg, 0.53 mmol), Ac (19 mg, 0.27 mmol), 4-cyanopentanoic acid dithiobenzoate (CTP) (7.42 mg, 2.66 × 10^{-2} mmol) and 4,4′-azobis-4-cyanovaleric acid (ACVA) (2.98 mg, 1.06 × 10^{-2} mmol) ($[MEO2MA]_0/[MAAmBO]_0/[Ac]_0/[CTP]_0/[ACVA]_0$ = 170/20/10/1/0.4) were dissolved in 4 mL of methanol. After degassing with nitrogen gas for 30 min, the mixture was allowed to polymerize for 24 h at 60 °C. The P(MEO2MA86.7-*co*-MAAmBO4.8-*co*-Ac8.5) was purified by the same protocol as shown above.

3. Preparation of P(MEO2MA93.9-*co*-MAAmBO5.2-*co*-PyMA0.9)

MEO2MA (900 mg, 4.78 mmol), MAAmBO (116 mg, 0.53 mmol), PyMA (16 mg, 5.31 × 10^{-2} mmol), 4-cyanopentanoic acid dithiobenzoate (CTP) (7.42 mg, 2.66 × 10^{-2} mmol) and 4,4′-azobis-4-cyanovaleric acid (ACVA) (2.98 mg, 1.06 × 10^{-2} mmol) ($[MEO2MA]_0/[MAAmBO]_0/[PyMA]_0/[CTP]_0/[ACVA]_0$ = 178/20/2/1/0.4) were dissolved in 4 mL of methanol. After degassing with nitrogen gas for 30 min, the mixture was allowed to polymerize for 24 h at 60 °C. The P(MEO2MA93.9-*co*-MAAmBO5.2-*co*-PyMA0.9) was purified by the same protocol as shown above.

4. Preparation of P(MEO2MA99.0-*co*-PyMA1.0)

MEO2MA (990 mg, 5.26 mmol), PyMA (16 mg, 5.31 × 10^{-2} mmol), 4-cyanopentanoic acid dithiobenzoate (CTP) (7.42 mg, 2.66 × 10^{-2} mmol) and 4,4′-azobis-4-cyanovaleric acid (ACVA) (2.98 mg, 1.06 × 10^{-2} mmol) ($[MEO2MA]_0/[PyMA]_0/[CTP]_0/[ACVA]_0$ = 198/2/1/0.4) were dissolved in 4 mL of methanol. After degassing with nitrogen gas for 30 min, the mixture was allowed to polymerize for 24 h at 60 °C. The P(MEO2MA99.0-*co*-PyMA1.0) was purified by the same protocol as shown above.

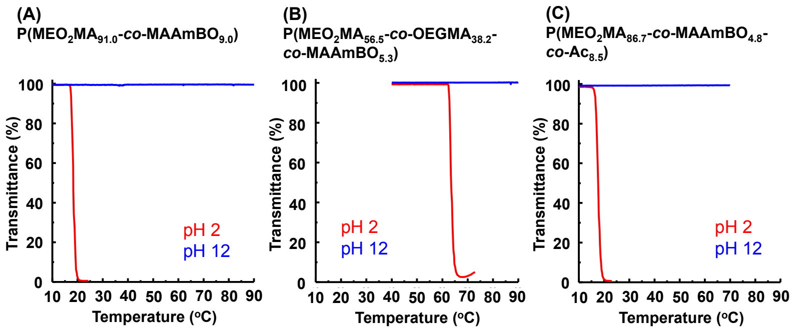

Figure S1. Transmittance changes of 0.1 wt % (**A**) P(MEO$_2$MA$_{91.0}$-*co*-MAAmBO$_{9.0}$), (**B**) P(MEO$_2$MA$_{56.5}$-*co*-OEGMA$_{38.2}$-*co*-MAAmBO$_{5.3}$) and (**C**) P(MEO$_2$MA$_{86.7}$-*co*-MAAmBO$_{4.8}$-*co*-Ac$_{8.5}$) at pH 2 (red line) and 12 (blue line) as a function of temperature.

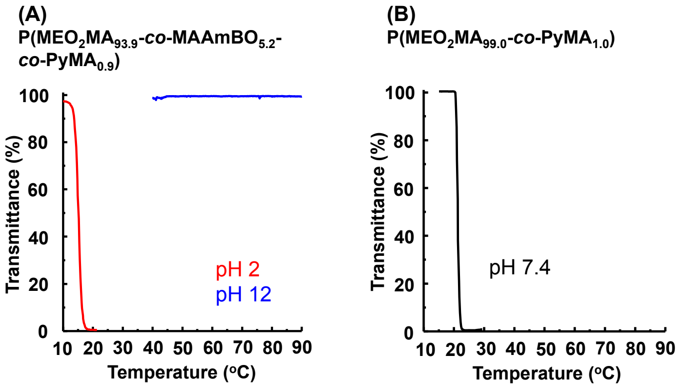

Figure S2. (**A**) Transmittance changes of 0.1 wt % P(MEO2MA93.9-*co*-MAAmBO5.2-*co*-PyMA0.9) at pH 2 (red line) and 12 (blue line) as a function of temperature. (**B**) Transmittance changes of 0.1 wt % P(MEO2MA99.0-*co*-PyMA1.0) at pH 7.4 PBS as a function of temperature.

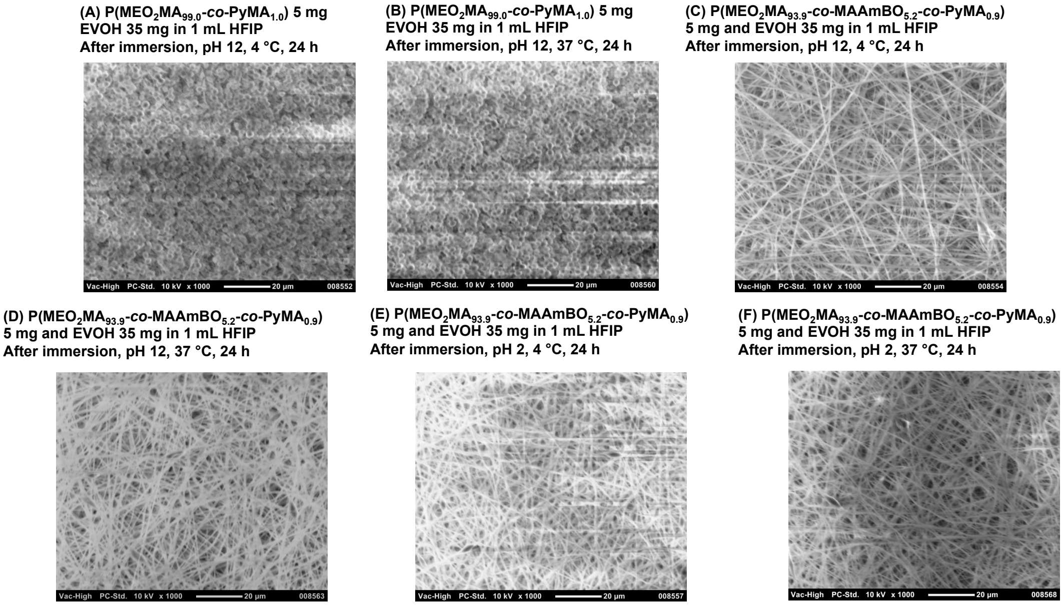

Figure S3. SEM images of electro-spinning samples after immersion for 24 h at various conditions: (**A**) P(MEO2MA99.0-*co*-PyMA1.0)/EVOH, pH 12, 4 °C; (**B**) P(MEO2MA99.0-*co*-PyMA1.0)/EVOH, pH 12, 37 °C; (**C**) P(MEO2MA93.9-*co*-MAAmBO5.2-*co*-PyMA0.9)/EVOH, pH 12, 4 °C; (**D**) P(MEO2MA93.9-*co*-MAAmBO5.2-*co*-PyMA0.9)/EVOH, pH 12, 37 °C; (**E**) P(MEO2MA93.9-*co*-MAAmBO5.2-*co*-PyMA0.9)/EVOH, pH 2, 4 °C; and (**F**) P(MEO2MA93.9-*co*-MAAmBO5.2-*co*-PyMA0.9)/EVOH, pH 2, 37 °C.

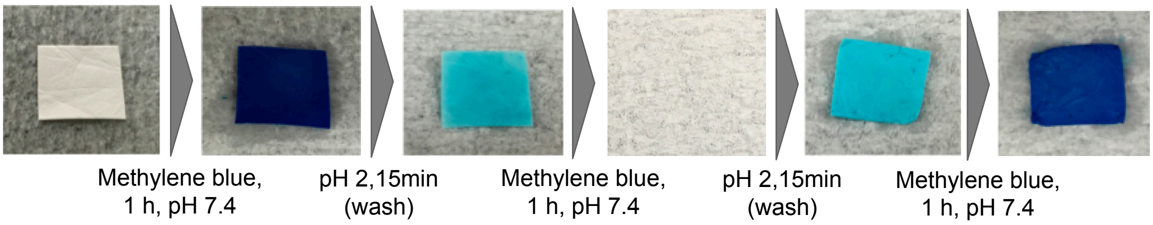

Figure S4. Reusability test of EVOH/P(MEO2MA86.7-*co*-MAAmBO4.8-*co*-Ac8.5) nanofiber on pH-responsive adsorption/desorption of methylene blue dye.

© 2016 by the authors; licensee MDPI, Basel, Switzerland. This article is an open access article distributed under the terms and conditions of the Creative Commons by Attribution (CC-BY) license (http://creativecommons.org/licenses/by/4.0/).
